# Considering the Validity of the SAIL Trial—A Navel Gazers Guide to the SAIL Trial

**DOI:** 10.3389/fped.2019.00495

**Published:** 2019-11-27

**Authors:** Haresh Kirpalani, Martin Keszler, Elizabeth E. Foglia, Peter Davis, Sarah Ratcliffe

**Affiliations:** ^1^Division of Neonatology, Children's Hospital of Philadelphia, University of Pennsylvania, Philadelphia, PA, United States; ^2^Department of Pediatrics, Warren Alpert Medical School, Women and Infants Hospital of Rhode Island, Brown University, Providence, RI, United States; ^3^Newborn Research Centre, The Royal Women's Hospital, Melbourne, VIC, Australia; ^4^Division of Biostatistics, Department of Public Health Sciences, University of Virginia, Charlottesville, VA, United States

**Keywords:** newborn, preterm, sustained inflation, clinical trial, methodological recommendations, evidence based practice, delivery, resuscitation

## Abstract

This article explores the validity of the Sustained Aeration Inflation for Infant Lungs (SAIL) randomized controlled clinical trial. The SAIL trial enrolled 460 infants out of a planned 600, but the trial was stopped early for harm. We ask here, whether there were any threats to validity in the trial as conducted. We then explore what design elements of the trial could have been improved upon. Finally, we consider what the implications are for future trials in this arena.

**Clinical Trial Registration:** www.clinicaltrials.gov, Identifier: NCT02139800

## Background

The Sustained Aeration for Infant Lungs (SAIL) trial^1^ asked the PICOT question: “In preterm infants born at 23+0 to 26+6 weeks gestation, who required resuscitation, did the randomized intervention of sustained inflation (SI) followed by nCPAP, as compared to standard resuscitation (nCPAP followed if needed by intubation) reduce the primary outcome of BPD or death by 36 weeks?” (1). This was an appropriate question following extensive animal work (2, 3), and some human research, suggesting a potential benefit of SI (4–8). Two systematic reviews of the human studies concluded a larger trial was needed (9, 10). Various authoritative bodies also recommended performing larger trials before SI could be considered standard practice (11, 12).

In the 18 SAIL participating sites in 9 countries, two procedures to obtain consent were used. In 16 sites, women likely to deliver within the gestational age window were approached for antenatal consent, unless delivery was imminent. With IRB approval, 4 of these sites endorsed a deferred consent process if there was insufficient time for an antenatal consent, or maternal condition made antenatal consent inappropriate; the IRBs of 2 sites approved deferred consent only (1). Details can be found in the report (1), and the study protocol (13).

As we reported (1), the trial was closed early for harm (excess early mortality) in the SI group, and very low probability of benefit with respect to the primary outcome if enrollment continued. Prior to trial start, the Data Safety Monitoring Board, trial sponsors (NICHD) and executive members of the trial, agreed on stopping rules for efficacy. In addition, an extensive early reporting system of harms was initiated. Early deaths were defined as those occurring within the first 48 h of life. Monitoring of this and other adverse outcomes was pre-planned.

The trial was stopped after the enrolment of 460 of the planned sample size of 600 infants. Our abstract reports that: “Among 460 infants randomized (mean [SD] gestational age, 25.30 [0.97] weeks; 50.2% female), 426 infants (92.6%) completed the trial. In the sustained inflation group, 137 infants (63.7%) died or survived with BPD vs. 125 infants (59.2%) in the standard resuscitation group (adjusted risk difference [aRD], 4.7% [95% CI, −3.8% to 13.1%]; *P* = 0.29). Death at <48 h of age occurred in 16 infants (7.4%) in the sustained inflation group vs. 3 infants (1.4%) in the standard resuscitation group (aRD, 5.6% [95% CI, 2.1–9.1%]; *P* = 0.002). Blinded adjudication detected an imbalance of rates of early death possibly attributable to resuscitation (sustained inflation: 11/16; standard resuscitation: 1/3). Of 27 secondary efficacy outcomes assessed by 36 weeks' postmenstrual age, 26 showed no significant difference between groups” (1). Moreover, this excess early death was most prominent in the pre-specified stratum containing the smallest and most immature infants. Of 16 early deaths in the sustained inflation group, 11 were in the 23- to 24-week stratum; and of 3 deaths in the standard resuscitation group, 2 were in the 23- to 24-week stratum.

When the Sustained Aeration for Lung injury (SAIL) trial was closed in January 2018 for harm, it was followed by considerable soul searching by the investigators. Could the results have been anticipated? Should the study have been prematurely closed? How were the results to be interpreted? Following publication, these questions were quickly echoed by the neonatal community. In this paper, we address two questions: (i) How valid were the results of the SAIL trial, and thus how confident can we be about these results? (ii) Could the design of the trial have been improved? As a corollary, we also ask if there are the implications for future study design.

### The Validity of the SAIL Trial

The validity, or “believability” of a trial is composed of two parts—internal and external validity (14). Internal validity asks how well a trial was conducted, in ensuring appropriate safeguards against bias, or systematic deviations from the truth? (15). In particular, were randomization and trial methods adequate to minimize threats of bias, which might render baseline risk factors between groups unequal (16). External validity addresses whether the results are generalizable to the wider population, and asks “Do I as a clinician recognize these infants, and could I carry out the maneuver of interest in these infants?”

Internal validity, judged by scrutiny of demographic baseline variables, appears to carry no threats of bias. In particular, there were no imbalances of gender, birth weights, or receipt of antenatal corticosteroids (1). However, one center exclusively used a deferred consent process. Overall, 34 randomized infants from the deferred consent group, were subsequently excluded because of lack of parental consent. Overall, there was no imbalance in those withdrawing consent by group: the CONSORT diagram (1) depicts 21/114 SI group vs. 13/111 standard resuscitation group [*P* = 0.19 unadjusted RD 13.1% (−4.7, 30.9); by Fisher exact]. Nonetheless, early stopping did constitute a possible threat to internal validity (17). Further potential concerns regarding validity arise because we did not monitor at the bedside the adherence to the SI ranges chosen or the effectiveness with which the maneuver was performed. However, this would have been extremely expensive to do, and video recording would not have been accepted by some IRBs. Moreover, an extensive training programme was undertaken. Finally, results analyzed by geographical regions showed no heterogeneity i.e., there was no evidence of a differential effect on primary outcome between sites where SI had been standard practice (Europe) compared to previously naïve sites (North America and Australia).

External validity appears satisfactory since the population of 23–26 week gestation infants is likely to be the same across well-resourced countries. However it is true that SAIL inclusion criteria specified: “Infants between 23 weeks 0 days' and 26 weeks 6 days' gestational age were eligible if they required positive pressure resuscitation because of inadequate respiratory effort or a heart rate <100 beats per minute (bpm).” This could be termed a “rescue” approach, and differs from other studies which enrolled all very small preterm infants, where a “prophylactic” approach was used (5).

This may explain a lack of benefit from SI in the SAIL trial and is consistent with the observation in a rabbit model that effective sustained inflation requires an open glottis (18). In addition, some human studies suggest that gain in Functional Residual Capacity is only seen when the infant is actively breathing (19, 20). Nonetheless, in the SAIL CONSORT diagram, of 546 infants assessed for eligibility in SAIL, only 86 were excluded as ineligible on delivery, of whom 70 had adequate respiration. Therefore, most infants in this GA category [mean (SD) 25.3 (0.97) weeks] were not judged to have adequate respirations at birth. This contrasts with video observations where most preterm infants, showed spontaneous respirations (21). However, these infants were more mature [mean (SD) GA of 26(2) week]. There is a higher vulnerability of the most immature infants in SAIL, such that most of the deaths are in the 23–24 week gestation. One implication of this finding is that caution should always be exercised in extrapolating findings from a more mature population to infants at the borderline of viability who may respond differently or be more vulnerable to adverse effects of an intervention.

One other feature of external validity is whether the results make clinical sense. Thus, while a difference in rates of early death was evident, the causes of this early death were adjudicated by the site investigators. There was no mandated post-mortem. No clear causal pathways mediating this excess of early death are obvious. Prior to conducting the trial, either pneumothorax or excess intraventricular hemorrhage would have been amongst the predicted causal pathways, but neither were elevated in the SI group (1). While this is intellectually unsatisfactory, it is counter-weighed by the undoubted numerical excess of death in the first 48 h of life.

Having ruminated on these issues, there may be no agreement amongst observers. Perhaps all would agree that the most robust assessment of the validity of the findings of harm would be replication in a similar large trial. Or failing that, a secondary confirmation from pooled data of gestational age subgroups. Such a study has been performed, by Foglia and colleagues, and is currently under review.

### Trial Design Implications

We suggest that there are at least three potentially actionable implications arising from the above considerations:

- The increasing awareness of extreme vulnerability at lower GAs would argue strongly for more attention to gestational age stratification in future studies. However, to avoid further frustrations, comparisons within strata should be adequately powered to be able to address substantive questions.
- DSMBs should not only pre-specify a priori statistical significance levels for efficacy, but also for harm. While mortality in the first 48 h was a pre-specified safety outcome reported to the DSMB, stopping rules were not defined for all reportable safety outcomes. Most of the investigating team believe that the trial should have been stopped, because ultimately the imbalance in early mortality rates represented a true and important difference in treatment effect rather than a chance occurrence. However, pre-specifying statistical significance levels, levels to trigger early stopping for defined safety outcomes might have made the process easier.
- Death is a common outcome in these vulnerable infants. Perhaps inadequate attention has been paid to the causes of death. Certainly one commentator has observed this to be a relatively important failing of recent neonatal trials (22).

## Conclusion

We are collectively still learning how best to perform trials in the delivery room. But the SAIL trial has taught us some important lessons, and we believe these should inform the next generation of newborn resuscitation trials. We suggest that as Sustained Inflation was applied in the SAIL trial, and in the dosing given in SAIL, the human experience differs from the animal data. To this extent, the maneuver of sustained inflation is another example where human and animal results of therapy differ (23). However, this is not equivalent to rejecting the concepts underlying sustained inflation, involving inflationary opening of the lung. New trials are underway to evaluate incremental PEEP in the delivery room (24). We are not aware of any trials being undertaken where an SI is delivered via an endotracheal tube, but since this is the modality used in the animal studies, such a paradigm shift may be indicated. In any case, the principle of using large randomized trials to assess delivery room interventions, should not be discarded despite their difficulty.

## Data Availability Statement

No datasets were generated or analyzed for this paper. However the SAIL datasets will be available subject to NICHD regulations, and approval by study executives - after a followup phase is completed.

## Author Contributions

All authors listed have made a substantial, direct and intellectual contribution to the work, and approved it for publication.

### Conflict of Interest

The authors declare that the research was conducted in the absence of any commercial or financial relationships that could be construed as a potential conflict of interest.

## Footnotes

^1^The full list of members participating in the SAIL trial are listed in the original publication (1).

## References

[1] Kirpalani H Ratcliffe SJ Keszler M Davis PG Foglia EE Te Pas A. Intermittent positive pressure ventilation on bronchopulmonary dysplasia or death among extremely preterm infants. The SAIL randomized clinical trial. JAMA. (2019) 321:1165–75. 10.1001/jama.2019.1660 30912836 PMC6439695

[2] te Pas AB Siew M Wallace MJ Kitchen MJ Fouras A Lewis RA. Establishing functional residual capacity at birth: the effect of sustained inflation and positive end-expiratory pressure in a preterm rabbit model. Pediatr Res. (2009) 65:537–41. 10.1203/PDR.0b013e31819da21b 19190537

[3] Sobotka KS Hooper SB Allison BJ Te Pas AB Davis PG Morley CJ. An initial sustained inflation improves the respiratory and cardiovascular transition at birth in preterm lambs. Pediatr Res. (2011) 70:56–60. 10.1203/PDR.0b013e31821d06a1 21659961

[4] te Pas AB Walther FJ. Trial of delivery-room respiratory management in very preterm infants. Pediatrics. (2007) 120:322–9. 10.1542/peds.2007-0114 17671058

[5] Lista G Boni L Scopesi F Mosca F Trevisanuto D Messner H SLI Trial Investigators. Sustained lung inflation at birth for preterm infants: a randomized clinical trial. Pediatrics. (2015) 135:e457–64. 10.1542/peds.2014-1692 25624390

[6] Harling AE Beresford MW Vince GS Bates M Yoxall CW. Does sustained lung inflation at resuscitation reduce lung injury in the preterm infant? Arch Dis Child Fetal Neonatal Ed. (2005) 90:F406–10. 10.1136/adc.2004.059303 15863490 PMC1721927

[7] Lindner W Högel J Pohlandt F. Sustained pressure-controlled inflation or intermittent mandatory ventilation in preterm infants in the delivery room? a randomized, controlled trial on initial respiratory support via nasopharyngeal tube. Acta Paediatr. (2005) 94:303–9. 10.1080/08035250410023647 16028648

[8] Jiravisitkul P Rattanasiri S Nuntnarumit P. Randomised controlled trial of sustained lung inflation for resuscitation of preterm infants in the delivery room. Resuscitation. (2017) 111:68–73. 10.1016/j.resuscitation.2016.12.003 27987395

[9] Schmölzer GM Kumar M Aziz K Pichler G O'Reilly M Lista G. Sustained inflation versus positive pressure ventilation at birth: a systematic review and meta-analysis. Arch Dis Child Fetal Neonatal Ed. (2015) 100:F361–8. 10.1136/archdischild-2014-306836 25550472

[10] Bruschettini M O'Donnell CP Davis PG Morley CJ Moja L Zappettini S. Sustained versus standard inflations during neonatal resuscitation to prevent mortality and improve respiratory outcomes. Cochrane Database Syst Rev. (2017) 7:CD004953. 10.1002/14651858.CD004953.pub3 28707404 PMC6483306

[11] Wyllie J Bruinenberg J Roehr CC Rüdiger M Trevisanuto D Urlesberger B. European Resuscitation Council Guidelines for Resuscitation 2015, section 7: resuscitation and support of transition of babies at birth. Resuscitation. (2015) 95:249–63. 10.1016/j.resuscitation.2015.07.029 26477415

[12] Wyckoff MH Aziz K Escobedo MB Kapadia VS Kattwinkel J Perlman JM Part13: neonatal resuscitation: 2015 American Heart Association guidelines update for cardiopulmonary resuscitation and emergency cardiovascular care. Circulation. (2015) 132 (Suppl. 2):S543–60. 10.1161/CIR.0000000000000267 26473001

[13] Foglia EE Owen LS Thio M Ratcliffe SJ Lista G Te Pas A. Sustained Aeration of Infant Lungs (SAIL) Trial: study protocol for a randomized controlled trial. Trials. (2015) 16:95. 10.1186/s13063-015-0601-9 25872563 PMC4372179

[14] Brian Haynes R Sackett DL Guyatt GH Tugwell P Clinical Epidemiology. How to Do Clinical Practice Research. 3rd ed. Philadelphia, PA: Lippincott Williams & Wilkins (2006). p. 8.

[15] Guyatt G Rennie D Meade MO Cook DJ JAMA Users' Guides to the Medical Literature. 2nd ed. New York, NY: McGraw-Hill (2008). p. 62.

[16] Kirpalani H. Control of bias has been recognized as important for a long time - but are we finally in control? Neonatology. (2019) 116:185–7. 10.1159/000500603 31280265

[17] Montori VM Devereaux PJ Adhikari NK Burns KE Eggert CH Briel M. Randomized trials stopped_**early**_for benefit: a systematic review. JAMA. (2005) 294:2203–9. 10.1001/jama.294.17.2203 16264162

[18] Crawshaw JR Kitchen MJ Binder-Heschl C Thio M Wallace MJ Kerr LT. Laryngeal closure impedes non-invasive ventilation at birth. Arch Dis Child Fetal Neonatal Ed. (2018) 103:F112–9. 10.1136/archdischild-2017-312681 29054974 PMC5868244

[19] van Vonderen JJ Hooper SB Hummler HD Lopriore E te Pas AB. Effects of a sustained inflation in preterm infants at birth. J Pediatr. (2014) 165:903–8.e1. 10.1016/j.jpeds.2014.06.007 25039041

[20] van Vonderen JJ Lista G Cavigioli F Hooper SB te Pas AB. Effectivity of ventilation by measuring expired CO_2_ and RIP during stabilisation of preterm infants at birth. Arch Dis Child Fetal Neonatal Ed. (2015) 100:F514–8. 10.1136/archdischild-2014-307412 26187933

[21] O'Donnell CP Kamlin CO Davis PG Morley CJ. Crying and breathing by extremely preterm infants immediately after birth. J Pediatr. (2010) 156:846–7. 10.1016/j.jpeds.2010.01.007 20236659

[22] Jobe AH. Unanticipated deaths in randomized controlled trials of very premature infants. J Pediatr. (2019). 10.1016/j.jpeds.2019.06.069. [Epub ahead of print]. 31378520

[23] Perel P Roberts I Sena E Wheble P Briscoe C Sandercock P. Comparison treatment effects between animal experiments and clinical trials: systematic review. BMJ. (2007) 334:197. 10.1136/bmj.39048.407928.BE 17175568 PMC1781970

[24] Tingay D Does the use of a high, dynamic, positive end-expiratory pressure strategy to support the lung during stabilisation at birth, compared with a standard, static PEEP, reduce the rate of death or bronchopulmonary dysplasia (BPD); and/or the rate of failure of non-invasive respiratory support in the first 72 hours after birth? Trial Registration: ACTRN12618001686291p.

